# Effect of Pulsed Electric Field Pretreatment on the Concentration of Lipophilic and Hydrophilic Compounds in Cold-Pressed Grape Seed Oil Produced from Wine Waste

**DOI:** 10.3390/foods13142299

**Published:** 2024-07-22

**Authors:** Natka Ćurko, Katarina Perić, Tomislava Vukušić Pavičić, Sandra Balbino, Marina Tomašević, Damir Iveković, Ivana Radojčić Redovniković, Karin Kovačević Ganić

**Affiliations:** Faculty of Food Technology and Biotechnology, University of Zagreb, Pierottijeva 6, 10000 Zagreb, Croatia; natka.curko@pbf.unizg.hr (N.Ć.); katarina.peric@pbf.unizg.hr (K.P.); tomislava.vukusic.pavicic@pbf.unizg.hr (T.V.P.); marina.tomasevic@pbf.unizg.hr (M.T.); damir.ivekovic@pbf.unizg.hr (D.I.); ivana.radojcic.redovnikovic@pbf.unizg.hr (I.R.R.); karin.kovacevic.ganic@pbf.unizg.hr (K.K.G.)

**Keywords:** grape pomace, sterols, tocochromanols, phenolic compounds, fatty acids, ORAC

## Abstract

Pretreatment of grape pomace seeds with a pulsed electric field (PEF) was applied to improve the extraction yield of cold-pressed grape seed oil. The effects of different PEF conditions, electric field intensities (12.5, 14.0 and 15.6 kV/cm), and durations (15 and 30 min) on the oil chemical composition were also studied. All PEF pretreatments significantly increased the oil yield, flow rate and concentration of total sterols (*p* < 0.05). In addition, similar trends were observed for total tocochromanols and phenolic compounds, except for PEF pretreatment under the mildest conditions (12.5 kV/cm, 15 min) (*p* < 0.05). Notably, the application of 15.6 kV/cm for 30 min resulted in the highest relative increase in oil yield and flow rate (29.6% and 56.5%, respectively) and in the concentrations of total tocochromanols, nonflavonoids, and flavonoids (22.1%, 60.2% and 81.5%, respectively). In addition, the highest relative increase in the concentration of total sterols (25.4%) was achieved by applying 12.5 kV/cm for 30 min. The fatty acid composition of the grape seed oil remained largely unaffected by the PEF pretreatments. These results show that PEF pretreatment effectively improves both the yield and the bioactive properties of cold-pressed grape seed oil.

## 1. Introduction

Global environmental issues have increased the need to introduce technologies and practices that can efficiently reduce the amount of waste generated in the food and agricultural sector and support the concepts of a circular economy and zero-emission philosophy. These concepts are also accepted by the winemaking sector, which focuses on sustainable wine production, and appropriate waste management that promotes the production of energy or waste valorization, particularly through the creation of new value-added products [1]. According to the International Organization of Vine and Wine, wine grape production in 2022 was estimated at 34.1 million tons, with wine production reaching 258 million hectoliters [2]. Grape wine pomace consists of skins and seeds and is obtained after grapes pressing during the winemaking process. Quantitatively and qualitatively it represents the most important fraction of wine waste, that accounts for about 20–25% of the weight of processed grapes, and contains high concentrations of various bioactive compounds [3,4]. In addition, both pomace skins and seeds are rich sources of hydrophilic phenolic compounds, as about 60% of these compounds remain in grape pomace, meaning that they are not extracted into wine during the winemaking process [4]. Lipophilic compounds, on the other hand, are predominantly found in seeds, where average oil content is around 15%, although this value can range from 7% to 22% [5,6]. In the last two decades, grape pomace skins and seeds have attracted considerable attention primarily from the perspective of phenolic compounds [4], while the mechanisms that improve the extraction of oil from grape pomace seeds, as well as their impact on the quality of oil, have been much less studied. Additionally, the popularity of grape seed oil is rising because it is derived from wine waste, thereby reducing waste, and also due to the extending application of this oil in pharmaceutical or food industry [5,7]. 

Grape seed oil is characterized by a high concentration of unsaturated fatty acids (about 90%), particularly linoleic acid (61.3–75.0%) and oleic acid (14.0–20.9%), while other fatty acids are present in lower amounts: palmitic (6.3–11.6%), stearic (3.5–6.6%), linoleic (0.2–1.8%), and arachidic acid (0.1–1.7%) [7,8,9]. This oil is a rich source of vitamin E active compounds, tocopherols and tocotrienols, which have strong antioxidant and biological activity and protect oil from negative oxidative changes and protect cells from oxidative stress. Namely, this oil contains about 63 to 1208 mg/kg of vitamin E active compounds (average 500 mg/kg), with α-tocopherol (<10–229 mg/kg) and α- and γ-tocotrienol (<10–538 and <10–785 mg/kg) as the main components [6,7,9,10]. Phytosterols that are lipophilic compounds of great biological importance due to their antioxidant activity and role in cholesterol metabolism were found at concentrations of 2576–11251 mg/kg, with the most abundant being β-sitosterol (835–8235 mg/kg; around 70%), followed by stigmasterol (285–961 mg/kg) and campesterol (128–944 mg/kg) [7,8,10]. The concentration of phenolic compounds in grape seed oil ranges from 23.9 to 115.5 mg/kg, with procyanidin B1, (+)-catechin, and (−)-epicatechin being the most important individual phenolic compounds [10,11]. In addition, grape seed oil is particularly interesting due to its vinous and fruity aroma [6,12]. However, these sensory properties are primarily expressed in cold-pressed grape seed oils—in contrast to solvent-extracted refined grape seed oils, which were found to be more neutral in taste and smell—and characterized by a lower content of bioactive compounds [6,13]. Extraction by cold pressing is recognized as an environmentally friendly technology because it does not require the use of toxic organic solvents or high energy consumption. However, cold pressing requires the use of high-quality material and results in an oil extraction yield that is usually not higher than 70% [6,14,15]. The yield of oil extraction can be increased by applying different pretreatments. However, since cold pressing does not involve heat or chemical pretreatments, pulsed electric field (PEF) as a nonthermal pretreatment is given an advantage over mechanical, chemical, or other thermal pretreatments in this context. 

PEF consists of the application of short, high-power electrical pulses (ms or µs), which lead to the generation of transmembrane potential when delivered to biological cells, due to the dielectric nature of the cell membrane [16,17]. The electromechanical stress generated within the membrane results in irreversible electroporation when the cells are exposed to an external electric field above the “critical” values. An increase in the porosity of the cell membrane will further lead to an increase in permeability, mass transfer during the extraction process and the extractability of various bioactive compounds [16]. Moreover, apart from improved extraction yield, the principal benefits of PEF compared to conventional processes also include an increase in the extraction rate, a reduction in the intensity of conventional extraction parameters, and a reduction in the energy cost of conventional extraction methods [18]. Interestingly, PEF showed a more selective extraction effect on the grape pomace bioactive compounds compared to other emerging technologies, such as ultrasound or high voltage electrical discharges [19].

Previous studies conducted on oilseeds (rapeseed, sunflower seeds, sesame, black cumin), olives and soybeans have shown that the application of PEF prior to oil extraction can increase extraction yields while maintaining overall oil quality [15,20,21,22,23,24,25]. A study conducted on black cumin seeds showed that the application of PEF pretreatment (3.25 kV/cm, 30 pulses and 20 µs) can significantly increase the extraction yield of cold-pressed black cumin oil (a relative increase of ~35%), oil density, oxidative stability, and extraction of phenolic compounds [20]. A relative increase of 4.6% in extraction yield compared to that of the control was found when PEF pretreatment (40 kV, 20 kV/cm, 0.5 Hz, 10 µs; 40 kJ/kg) was applied prior to cold pressing of sesame seeds [21]. Additionally, PEF (2 kV/cm; 25 Hz; 11.25 kJ/kg) was found to be a suitable technology to increase the extraction yield of virgin/extra virgin olive oil, resulting in 13.3% higher yields and significantly higher concentrations of total phenols, phytosterols, and tocopherols (1.5%, 9.9%, and 15.0%, respectively) [22]. Similarly, PEF pretreatments of different ranges (1–7 kV/cm, 30 and 80 pulses, 20 μs) were shown to be appropriate for cold-pressed rapeseed oil, particularly for increasing the extraction yield (4 kV/cm and 80 pulses; 10.24 kJ/kg) and concentration of phenolic compounds (7 kV/cm and 80 pulses; 31.36 kJ/kg) [15]. In addition, the application of different PEF parameters (1–7.0 kV/cm, 0.5–15 Hz, 10–120 s, 10–50 µs) to sunflower seeds showed that a maximum increase in extraction yield of 9.1% can be achieved when PEF (7.0 kV/cm; 15 Hz, 30 s, 30 µs) is combined with 40% solvent [23]. PEF pretreatment of hulled and non-hulled rapeseeds (5 kV/cm with 60 pulses and 7 kV/cm with 120 pulses; 30 µs) not only led to an increase in oil yield in cold-pressed and solvent-extracted oil but also to a higher concentration of total antioxidants, tocopherols, polyphenols and phytosterols [25]. Moreover, the beneficial effects of PEF treatment on the extraction of oil from olives and maize, isoflavonoids from soybeans, and phytosterols from maize germs have been demonstrated [24]. However, little is known about the effects of PEF on the grape seed oil extraction yield and chemical composition. This is because the effects of PEF depend not only on the intensity of the applied parameters (electric field intensity, pulse number, pulse duration/width, pulse frequency, etc.) but also on the structural tissue characteristics (cell size and shape, thickness of the cell wall), moisture, and oil content of the treated material [16,23,26]. In addition, it is important to note that grape seeds are characterized by relatively hard and rough structure, as the cell walls of the medium integument (hard seed coat) become impregnated with lignin during ripening, which is responsible for the hardness, form, and firmness of the seeds [27]. In our previous study, we noted that the application of PEF to milled grape pomace seeds prior to supercritical CO_2_ extraction had a crucial role in increasing extraction yields by offering the possibility for more selective extraction, particularly of sterols and phenolic compounds [10].

The objective of this research was to investigate the potential of PEF pretreatment for overcoming the problem of low extraction yield in cold pressing (utilizing intact whole pomace seeds) and to examine the impact of this pretreatment on the quality of Graševina grape seed oil.

## 2. Materials and Methods

### 2.1. Chemicals

Acetonitrile, ethanol, n-hexane, methanol and 2-propanol were purchased from J.T. Baker (Deventer, The Netherlands). Fatty acid methyl ester (FAME) standard mixture was purchased from Supelco (Bellefonte, PA, USA). N,O-bis(trimethylsilyl)trifluoroacetamide with trimethylchlorosilane, silica gel F254 plates and α-tocopherol were purchased from Merck (Darmstadt, Germany). 2′-azobis(2-methylpropionamidine) dihydrochloride (AAPH), 5-α-cholestanol, diethyl ether, disodium hydrogen phosphate, fluorescein, 6-hydroxy-2,5,7,8-tetramethylchroman-2-carboxylic acid, isooctane, methyl-β-cyclodextrin, phenolic standards, potassium hydroxide, sodium carbonate, sodium dihydrogen phosphate, and sodium hydrogen sulfate monohydrate were purchased from Sigma-Aldrich (St. Louis, MO, USA).

### 2.2. Plant Material

This research was conducted with Graševina (*Vitis vinifera* L.) white grape pomace obtained from the Kutjevo d.d. winery (Kutjevo, Croatia). Grapes were harvested in technological maturity in late September, vintage 2022, and underwent vinification process for commercial wine production. An amount of around 300 kg of grape pomace was collected after grape pressing. The grape seeds, which constituted around 12% of grape pomace, were immediately separated from the remaining pomace, gently cleaned from the pulp, and homogenized prior to further processing and oil production.

### 2.3. Pulsed Electric Field (PEF) Pretreatment of Grape Pomace Seeds

Pretreatments were carried out in a PEF system (HVG60/1, Impel d.o.o., Zagreb, Croatia) assembled with a generator and a treatment unit, as previously described [28]. The static PEF chamber of the treatment unit consisted of two parallel electrodes separated by a distance of 32 mm. Prior to PEF pretreatment, the seeds were soaked in distilled water (1:1.5) for 4 h to reach a moisture content of 40%. A total of 1 kg of the soaked seeds was placed in the PEF chamber, and different pretreatments were applied (Table 1): pulse powers of 40, 45, and 50 kV, each for 15 and 30 min, with a pulse frequency of 100 Hz and a pulse width of 1 µs, were selected based on our previous study [10] and data from the literature [21,25]. Therefore, the six PEF samples obtained were named based on their different electric field strengths and pretreatment duration: 12.5 kV/cm for 15 min (PEF1_15) and 30 min (PEF1_30); 14 kV/cm for 15 min (PEF2_15) and 30 min (PEF2_30); and 15.6 kV/cm for 15 min (PEF3_15) and 30 min (PEF3_30). Each PEF pretreatment was conducted in triplicate. The total estimated specific energy inputs for the above samples were: 36.0 and 72.0 kJ/kg for PEF1_15 and PEF1_30, 45.6 and 91.1 kJ/kg for PEF2_15 and PEF2_30, and 56.3 and 112.5 kJ/kg for PEF3_15 and PEF3_30, respectively. The temperature measured before pretreatment was 20.0 ± 0.5 °C, while that measured after pretreatment ranged from 22.3 ± 0.1 °C for PEF1_15 to 23.9 ± 0.2 °C for PEF3_30.

### 2.4. Oil Extraction by Cold Pressing

Cold pressing was performed using a laboratory screw press (PR-F-100, Oilpressparts GmbH & Co., KG, Niederkrüchten, Germany). Prior to pressing, the grape pomace seeds were dried for 12 h at 35 °C in an dryer to standardize the moisture content of the samples to 8%, as this parameter affects the efficiency of pressing [19,23]. The oils were extracted by pressing 750 g of grape pomace seeds, without conditioning the seeds or applying heat [10]. The rotation speed of the screw press was set to 30 rpm, while a nozzle diameter of 10 mm was used. Cold pressing was conducted in triplicate. The obtained oils were centrifuged at 5000 rpm for 10 min and stored at −18 °C under nitrogen in dark glass bottles.

### 2.5. Oil Extraction Yield and Flow Rate

The grape seeds oil extraction yield was calculated as the percentage of oil extracted by cold pressing relative to the total extractable oil [14] according to Equation (1). The total extractable oil content in the grape pomace seeds was 10.32 ± 0.04%, as determined by the standard method [29]. In addition, the oil flow rate was calculated as the mass of extracted oil in the function of time [30] as presented in Equation (2).

(1) $$Oil\;yield\left(\%\right)=\frac{mass\;of\;oil\;extracted\;by\;cold\;pressing}{mass\;of\;total\;extratable\;oil\;in\;grape\;pomace\;seeds}⨯100$$

(2) $$Oil\;flow\;rate\left(g/h\right)=\frac{mass\;of\;oil\;extracted\;by\;cold\;pressing}{time}$$

### 2.6. Determination of Sterols by GC-FID/MS

Analysis of sterols was conducted on a GC-FID/MS instrument (6890N/5973 Inert, Agilent Technologies, Santa Clara, CA, USA) with the standard method [31]. The cleavage of bound phytosterols to free analogs was conducted by alkaline saponification after the addition of 5α-cholestanol as an internal standard. Unsaponifiable matter containing free sterols was eluted by solid-phase extraction on aluminum oxide with diethyl ether. Sterols were separated from the rest of the unsaponifiable matter by thin layer chromatography on a silica gel plate with a solution of hexane and diethyl ether. The sterol fraction obtained was further silylated. The separation was conducted on a capillary column (30 m × 0.32 mm, 0.25 µm) (DB-17MS, Agilent Technologies, Santa Clara, CA, USA) as described earlier [32]: injector temperature set at 290 °C, split ratio 13.3:1, helium as the carrier gas with a flow rate of 1.5 mL/min, temperature program from 180 °C to 270 °C with an increase of 6 °C/min and further kept at 270 °C for 30 min, detector temperature set at 250 °C. The mass parameters were as follows: transfer line temperature, 280 °C; ionization voltage, 70 eV; MS ion source, 230 °C; and quadrupole 150 °C, scanning from 30–550 (*m*/*z*). The sterols were identified using the NIST 17 library. The concentrations of sterols were expressed in mg/kg of the analyzed sample.

### 2.7. Determination of Tocochromanols by HPLC-FLUO

The analysis of tocopherols and tocotrienols was performed on an HPLC-FLUO system (1290 Infinity II LC, Agilent Technologies, Santa Clara, CA, USA) following the standard method [33]. The separation was conducted on a normal-phase silica column (250 mm × 4 mm, 5 µm) (LiChrosorb Si 60, Merck, Darmstadt, Germany) with hexane/2-propanol (99.3:0.7, *v*/*v*) as the mobile phase at a flow rate of 0.9 mL/min. Detection and identification of tocochromanols (α- and γ-tocopherols, α- and γ-tocotrienols and plastochromanol-8) were conducted as earlier described [14]. The concentrations of tocochromanols were expressed in mg/kg of the analyzed sample.

### 2.8. Spectrophotometric Determination of Total Phenols

Total phenols were analyzed with a UV-VIS spectrophotometer (Specord 50 PLUS, Analytik Jena, Jena, Germany) applying the Folin–Ciocalteu method [34]. The phenolic compounds were extracted with methanol/water (90:10, *v*/*v*) following previously proposed procedure [11]. The concentrations of total phenols were expressed in mg gallic acid equivalents (GAE)/kg of the analyzed sample.

### 2.9. Determination of Individual Phenolic Compounds by HPLC-DAD/MS

The analysis of the individual phenolic compounds was carried out on an HPLC-DAD/MS system (1290 Infinity II LC, Agilent Technologies, Santa Clara, CA, USA). Phenolic compounds were separated on a C18 column (250 mm × 4.6 mm, 5 µm) (Gemini, Phenomenex, Torrance, CA, USA), with mobile phase A consisting of water/formic acid (99.7:0.3, *v*/*v*) and mobile phase B consisting of methanol, according to the gradient detailed in our previous publication [10]. Detection was carried out at 280 nm for gallic acid, hydroxybenzoic acid, (+)-catechin, (−)-epicatechin and the procyanidin dimer B1; at 320 nm for *p*-coumaric and ferulic acid; and at 360 nm for quercetin and myricetin. The phenolic compounds were identified by comparing their retention times with those obtained by injection of standards, as well as the mass spectral data, and quantified using the method of external standards listed above. The concentrations of individual phenolic compounds were expressed in µg/kg of the analyzed sample.

### 2.10. Determination of Fatty Acid Profile by GC-FID

Analysis of the fatty acid profile was performed on a GC-FID instrument (6890N, Agilent Technologies, Santa Clara, CA, USA). Transmethylation was conducted following the standard method [35], and the corresponding methyl esters were determined according to the standard method [36]. Separation was conducted on a capillary column (60 m × 0.25 mm, 0.25 µm) (DB-23, Agilent Technologies, Santa Clara, CA, USA) according to earlier described parameters [32]: injector temperature set at 250 °C; split ratio 75:1, helium as the carrier gas with a flow rate of 0.7 mL/min; temperature program from 120 °C to 160 °C with a rise of 4 °C/min, continuing to 190 °C with a rise of 10 °C/min, where it was held for 30 min; and detector temperature set at 280 °C. The fatty acid methyl esters were identified by comparing their retention times with those obtained by injection of FAME standards. The content of each fatty acid is expressed as a percentage (%) of the total fatty acids.

### 2.11. Determination of Antioxidant Capacity

The antioxidant capacity was determined using the oxygen radical absorbance capacity (ORAC) method, following the protocol for both hydrophilic (H-ORAC) and lipophilic (L-ORAC) methods [8,37]. The H-ORAC and L-ORAC values were expressed in µmol Trolox Equivalents (TE)/g of the analyzed sample.

### 2.12. Scanning Electron Microscopy (SEM) Analysis

Microstructural analysis of the grape pomace seed surfaces was performed using a scanning electron microscope (Vega3 LMH, Tescan, Brno, Czech Republic). Control (untreated) and PEF-pretreated samples were analyzed and compared to observe the changes (pores) caused by the electroporation mechanism. Before SEM analysis, the samples were air-dried overnight at 60 °C, then fixed on SEM stubs with silver epoxy and sputter-coated with a thin layer of gold-palladium alloy. SEM was carried out under high vacuum at an accelerating voltage of 10 kV.

### 2.13. Data Analysis

The analyses were performed in triplicate, and the results are expressed as the mean value ± standard deviation. The statistical data analysis included analysis of variance (ANOVA) and Tukey’s HSD test which was applied as a comparison test when the samples differed significantly after ANOVA (*p* < 0.05) using Statistica v.14 software (Tibco Software Inc., Palo Alto, CA, USA).

## 3. Results and Discussion

Different PEF pretreatments were applied to the grape pomace seeds, and these seeds were further pressed to obtain cold-pressed grape seed oils (Table 1). The effects of PEF pretreatment on oil yield and flow rate, and chemical composition and antioxidant capacity were further studied. 

### 3.1. Oil Yield and Oil Flow Rate

**Figure 1:**
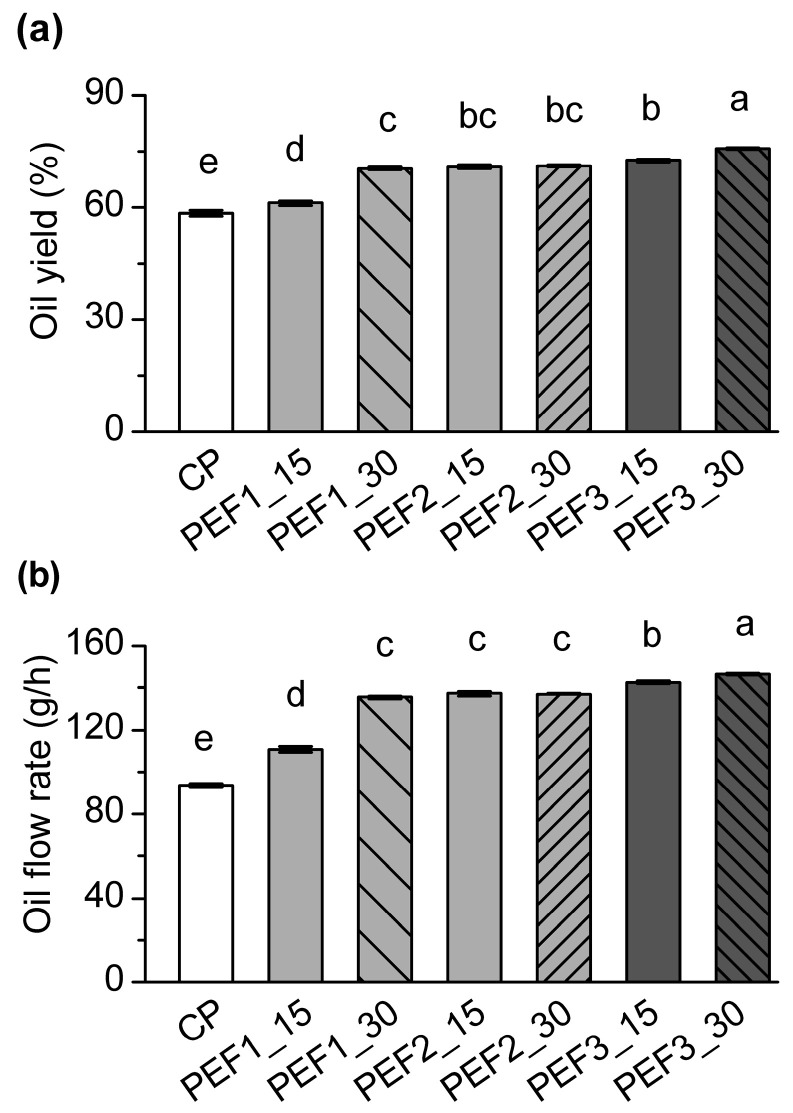
Effect of PEF pretreatment on the cold pressing process: (**a**) oil yield and (**b**) oil flow rate. Data are presented as mean value ± standard deviation over three replicates. ANOVA to compare data; different lowercase letters among column bars indicate significant differences between samples (Tukey’s test, *p* < 0.05).

The results presented in Figure 1 show the significant impact of PEF pretreatment on oil yield and flow rate, with similar trends observed for the two analyzed indicators of the pressing process. PEF pretreatments, compared to the control sample, significantly increased the oil extraction yield of cold pressing (Figure 1a) (*p* < 0.05). These were in accordance with earlier studies conducted on rapeseed, sesame seed, or olive oil pasta [15,21,22,25]. As earlier proposed [16], cells exposed to a critical PEF exhibit high transmembrane potential due to the accumulation of charge on the membrane surface. This further induces local changes in the cell membrane through the formation of pores, which increase cell permeability and mass transfer and enhance the extraction of oil and different components. 

The oil yield of the samples pretreated with PEF ranged from 61.2 to 75.7% (Figure 1a), which corresponds to a relative increase of 4.9 to 29.6% compared to the untreated control sample and is consistent with the literature [38]. Increasing the intensity of the electric field from 12.5 kV/cm to 15.6 kV/cm and a longer pretreatment duration contributed to significantly higher yields in both cases (*p* < 0.05). However, this was not found when pretreatments of 12.5 and 14.0 kV/cm were compared. An increase in the electric field intensity, pretreatment time/number of pulses or total amount of energy does not necessarily always result in higher oil recovery [15,24,25]. Namely, the outcomes of PEF pretreatment depend not only on the number of pores formed but also on the number of output ducts opened after pretreatment, which, in addition to the PEF intensity, is closely related to the structural characteristics of plant tissues [26] and should therefore be considered when selecting PEF parameters.

In addition, apart from affecting the yield, PEF pretreatments also shortened the pressing process and hence significantly increased the oil flow rate (Figure 1b) (*p* < 0.05). The values of the pretreated samples ranged from 110.7 to 146.4 g/h (Figure 1b), which corresponds to a relative increase of 18.3% to 56.5% compared to the untreated control sample, following similar trends for oil yield to those described above. Thus, the highest flow rate was found for a PEF pretreatment of 15.6 kV/cm and 30 min (*p* < 0.05).

### 3.2. Sterols

PEF pretreatments significantly increased the concentration of total sterols in all grape seed oils (*p* < 0.05), and the results showed 5.3 up to 25.4% higher values compared to the control sample (Table 2). Indeed, sterols play an important role as structural cell membrane components, where they regulate membrane fluidity and permeability in response to environmental stress, and are involved in the control of membrane-associated metabolic processes [39,40]. Thus, the disintegration of cell membranes and the formation of small pores caused by PEF pretreatment enhances the recovery of these compounds in the oil [24].

When comparing different extraction methods, it can be stated that the relative increase in sterol concentration was greater when PEF was combined with cold pressing (up to 25.4%) than when combined with other extraction methods, such as supercritical CO_2_ extraction [10] or conventional hexane extraction [25]. For instance, the application of 5 kV/cm at 120 Hz for 5 min on grape pomace seeds contributed to a relative increase of 8.5% in the oil extracted with supercritical CO_2_ (4926.7 to 5347.0 mg/kg) [10], while the application of 7 kV/cm and 120 pulses contributed to a relative increase of 3.6% (619 to 641 mg/100 g) and 2.8% (604 to 621 mg/100 g) in hulled and unhulled rapeseed extracted with hexane, respectively [25].

Considering the effects of PEF intensity and duration, different trends were observed for individual sterols, but the most important ones were reflected in the overall sum of total sterols (Table 2). The most prominent changes were found for the PEF of 12.5 kV/cm, for which increasing the pretreatment duration from 15 to 30 min caused a significant increase in the concentrations of β-sitosterol, stigmasterol and campesterol (*p* < 0.05). Since these sterols are the most abundant and account for more than 90% of the sterols in grape seed oil [7,8,10], the latter pretreatment (12.5 kV/cm and 30 min) proved to be the most effective in terms of total sterol contribution. As previously stated, the concentrations found in this pretreatment were 25.4% higher than those in the control (PEF untreated) cold-pressed oil (*p* < 0.05). On the other hand, PEF proved to be less effective for 14.0 kV/cm and particularly for 15.6 kV/cm compared to 12.5 kV/cm. Moreover, for 15.6 kV/cm, in contrast to the other PEF intensities tested, extending the treatment duration did not have a favorable effect on the concentration of total sterols, resulting in even lower concentrations after 30 min than after 15 min pretreatment (*p* < 0.05). This could not be attributed to the higher oil yield and potential “dilution” effect, as similar trends were obtained between samples when sterols concentrations in mg per kg of seeds were calculated (Table S1).

Furthermore, a parallel can be drawn with a previous study [24], where an electric field strength of 0.6 kV/cm to 7.3 kV/cm and 120 pulses applied to maize germs resulted in the highest phytosterol oil yield in the first (1039 mg/100 g) compared to the second pretreatment (900 mg/100 g). Although little is known about the mechanism behind the changes of sterols, it has been established that high intensity electric fields and/or duration of treatment can cause undesirable macroscopic changes in the structure of the membrane, and chemical changes, including oxidative degradation or other secondary chemical reactions involving food constituents such as phenolic compounds, lipids, vitamins, or proteins [17,41,42]. Since sterols are structural membrane components, further information on the effect of PEF on sterols is needed. In addition, sterols located in the shielding tissue surrounding the endosperm/embryo of grape seeds are likely to be primarily affected, and in some cases, such as sunflower seeds, the portion of these sterols can reach a non-negligible rate of 25% [43]. Nevertheless, in agreement with data from the literature [24,25], the present results indicate that an optimal phytosterol yield can be achieved only if the PEF parameters are adjusted to the characteristics of the pretreated plant tissue.

### 3.3. Tocochromanols

All pretreatments, except the mildest one (12.5 kV/cm and 15 min), contributed to significantly higher concentrations of tocochromanols compared to the control (*p* < 0.05), with an overall relative increase from 8.2 to 22.1% (Table 3). In accordance with the present results, previous studies have also demonstrated the beneficial effect of PEF pretreatment when joint with cold pressing or other extraction methods [10,22,25]. In particular, tocochromanols play an important role as lipophilic antioxidants in protecting the oil from oxidative damage during storage [44] and thus contribute to oil quality [22].

Increasing the intensity of the electric field and the duration of the treatment were found to be important PEF parameters affecting the concentrations of tocochromanols (Table 3). Significantly higher concentrations of total tocochromanols were obtained for samples pretreated with 14.0 kV/cm than for those pretreated with12.5 kV/cm, and particularly with 15.6 kV/cm. Moreover, the extension of the treatment duration from 15 to 30 min additionally contributed to these results (*p* < 0.05). This finding is also consistent with our earlier observations on grape seed oil extracted with PEF-assisted supercritical CO_2_, which also showed a significant increase in total tocochromanols with the extension of pretreatment [10]. 

The trends established in total tocochromanols were dominated by the most represented compounds, γ-tocotrienol, followed by α-tocotrienol and α-tocopherol, as previously found for grape seed oil [7,8]. Among these compounds, α-tocopherol appears to be particularly susceptible to PEF pretreatment, as both increasing the electric field intensity and/or the duration of PEF pretreatment significantly increased the concentration of this compound. This was not surprising since α-tocopherol was homogeneously distributed in all grape seed tissues, making this compound more accessible to the action of PEF [44]. Moreover, this finding is also consistent with our earlier observations [10]. In addition, a study conducted with oil paste showed that PEF pretreatment among the individual isomers (α-, β-, γ- and δ-tocopherol) significantly affected only α-tocopherol, increasing its value by 25% [22]. Nevertheless, it is interesting to note that increasing the intensity of the PEF electric field and/or the pretreatment time had no effect on the concentrations of γ-tocopherol and plastochromanol-8, although the values were significantly higher in all pretreated samples than in the control (*p* < 0.05). However, the application of 15.6 kV/cm, particularly for 30 min, significantly affected all three major tocochromanols (α-tocotrienol, γ-tocotrienol, and α-tocopherol), resulting in the highest concentrations of individual and total tocochromanols (*p* < 0.05).

### 3.4. Phenolic Compounds

In agreement with data from the literature for other plant materials [15,22,25], our results (Table 4) showed significantly higher concentrations of total and almost all individual phenolic compounds in PEF-pretreated samples, except in the case of the lowest PEF electric field intensity and time (*p* < 0.05). These PEF pretreatments contributed to the relative increase from 24.1 up to 60.2% and from 20.1 up to 81.5% of total nonflavonoids and flavonoids concentrations, respectively. 

Among phenolic compounds, the most abundant fraction was that of flavonoids, represented primarily by procyanidin B1 and (+)-catechin as two main compounds [10,45]. In addition, among all phenolic compounds, (−)-epicatechin, quercetin, and myricetin showed the highest relative increase in all treatments (*p* < 0.05). Additionally, increasing the intensity and duration of the PEF electric field resulted in a gradual and significant increase in the concentrations of total nonflavonoids and flavonoids (*p* < 0.05). Similar results regarding phenolic compounds and the electric field intensity and duration of PEF pretreatment have previously been reported [15,25]. More precisely, an increase in both intensity and duration was noticed for all phenolic acids (*p* < 0.05), in accordance with our previous study [10]. The exception was hydroxybenzoic acid, where a further increase of PEF pretreatment from 14.0 kV/cm and 30 min had no significant effect on the concentration of this compound. A similar trend was also observed for myricetin, where no significant difference was found between 14.0 and 15.6 kV/cm.

On the other hand, a PEF below 14.0 kV/cm and 30 min showed no effect on the concentration of (+)-catechin. However, despite some differences noticed for the mildest pretreatments (14.0 kV/cm) compared to the lowest (12.5 kV/cm) and the highest (15.6 kV/cm) pretreatments, the increase from 12.5 kV/cm to 15.6 kV/cm was responsible for the significant increase in the concentration of total and all individual phenolic compounds as well as for the highest concentrations of these compounds in the latter (*p* < 0.05). 

### 3.5. Fatty Acids

The effects of PEF pretreatments on the fatty acid composition of the cold-pressed grape seed oils are presented in Table 5. The PEF pretreatments had no significant effect on the composition of myristic, palmitoleic, oleic, linolenic, or arachidic acids. In addition, PEF-pretreated samples showed significantly lower portions of palmitic acid and higher portions of linoleic acid for PEF pretreatment of 15.6 kV/cm compared to the control (*p* < 0.05). Nevertheless, even in the case of the most abundant linoleic acid [7,8,10], these changes represented a relative change of less than 0.2%. Therefore, no differences were obtained in the total sum of saturated, monounsaturated, and polyunsaturated fatty acids among PEF-pretreated and control samples. These results collaborate with the findings of previous work, which also reported that PEF pretreatment, prior to cold pressing, can induce only minor changes in fatty acid composition [15,46].

### 3.6. Hydrophilic and Lipophilic Oxygen Radical Absorbance Capacity (H-ORAC and L-ORAC)

**Figure 2:**
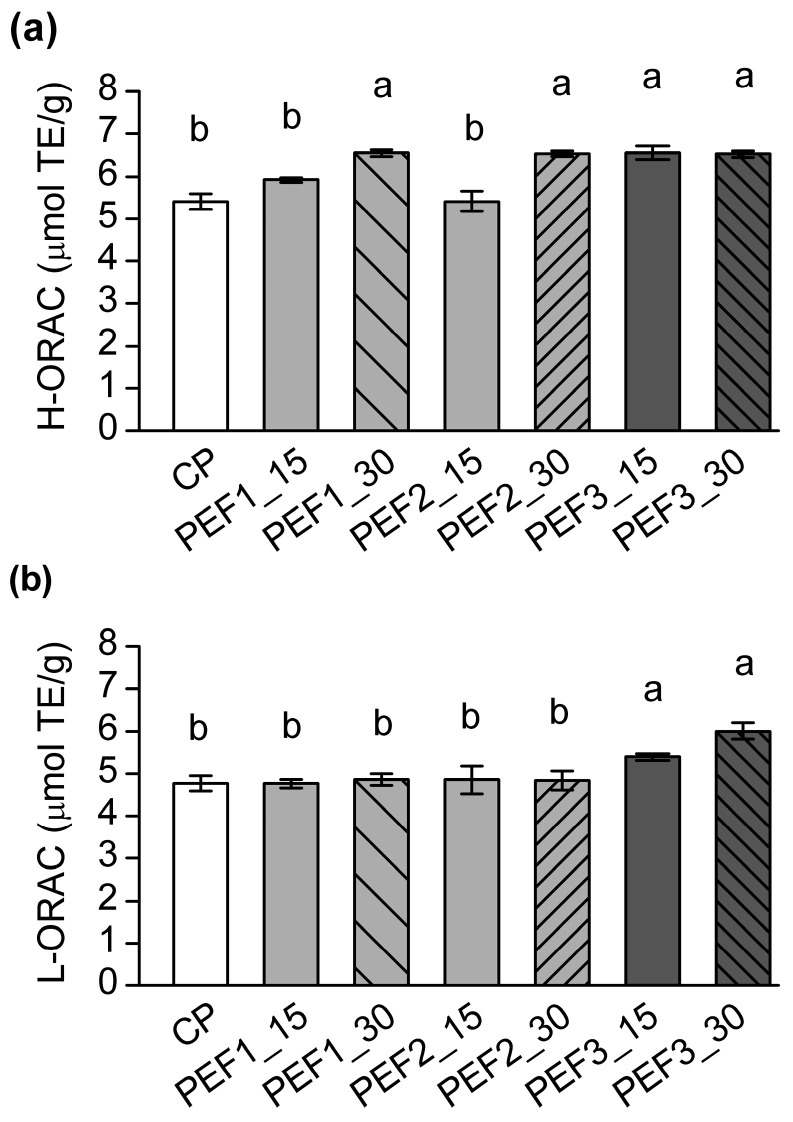
Effect of PEF and cold pressing on the antioxidant capacity of grape seed oil: (**a**) hydrophilic ORAC (H-ORAC) and (**b**) lipophilic ORAC (L-ORAC). Data are presented as mean value ± standard deviation over three replicates. ANOVA to compare data; different lowercase letters among column bars indicate significant differences between samples (Tukey’s test, *p* < 0.05).

The antioxidant capacities of grape seed oils, including H-ORAC and L-ORAC, were also affected by PEF pretreatments (Figure 2), primarily by increasing the intensity and/or duration of the higher electric fields. For example, significantly higher H-ORAC values were obtained for pretreatments of 12.5 and 14.0 kV/cm with 30 min duration, and 15.6 kV/cm with both 15 and 30 min (Figure 2a) (*p* < 0.05). However, the H-ORAC values of these pretreated samples were not significantly different from each other, corresponding to a relative increase of 13% compared to the control. Furthermore, L-ORAC appears to be less affected by lower intensity pretreatments compared to H-ORAC, as significantly higher values were only found for 15.6 kV/cm regardless of duration (Figure 2b) (*p* < 0.05). Nevertheless, these two PEF pretreatments (30 and 60 min) contributed to relative increases of 13% and 26%, respectively. These results obtained are in line with those of Guderjan et al. [25], who reported relative increases in antioxidant capacity of 11% and 13% in hexane extracted hulled and unhulled rapeseed oil, respectively. Our previous study also showed that the application of PEF prior to supercritical CO_2_ extraction can significantly affect hydrophilic fraction, with a relative increase in H-ORAC values of 7% up to 11%. In addition, the ranges obtained for ORAC values were in line with previous studies [8,9,10].

### 3.7. Surface Structure of Grape Pomace Seeds

**Figure 3:**
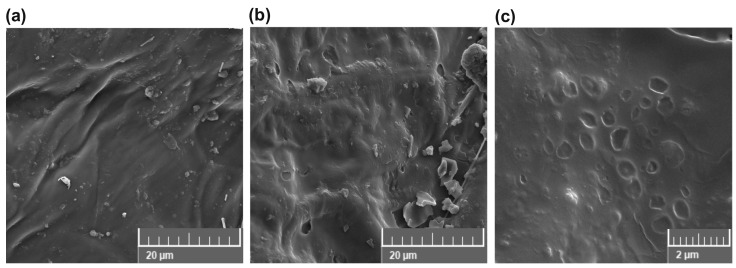
SEM images of grape pomace surface of the (**a**) control (untreated) sample, (**b**) PEF-pretreated (15.6 kV/cm, 30 min) sample, and (**c**) magnification of cavities induced by PEF pretreatment. Bar = 20 µm (**a**,**b**) and 2 µm (**c**).

The SEM images of the surface of grape pomace seeds revealed a markable difference between the untreated sample (control) (Figure 3a) and the PEF sample (PEF3_30) pretreated with 15.6 kV/cm for 30 min (Figure 3b,c). The PEF3_30 sample selected was the one exposed to the highest intensity and duration as well as the one most affected considering the oil extraction yield (Figure 1) and chemical composition (Table 2, Table 3, Table 4 and Table 5). The PEF pretreatments induced changes in the physical structure due to the formation of numerous pits and cavities (Figure 3b,c), even though grape seeds are generally characterized as seeds with a hard structure. Indeed, the structure of grape seeds comprises five zones: (i) cuticle and epidermis, (ii) outer integument, (iii) medium integument, (iv) inner integument, and (v) endosperm and embryo [27]. However, its hardness and roughness are mainly due to the intense lignification and dehydration of the outer integument (hard seed coat) that occurs during ripening [27]. Therefore, the upper zones—such as the cuticle, epidermis, and possibly the outer integument—are more affected by PEF pretreatment. In addition, the pits and cavities formed were predominantly round or spherical (Figure 3c), which is known to result from electroporation [20,23], and were found in a size range of 0.5–4 µm (Figure 3b,c). At the cellular level, electroporation is responsible for this increase in cell membrane porosity and leads to an increase in permeability and even rupture of the cell membrane [16]. This further enhances mass transfer during the extraction process and the extractability of valuable bioactive compounds [15,25,38]. This can also be supported with the results presented in Figure 2 and Figure 3 and Table 2, Table 3, Table 4 and Table 5.

Our study also revealed that among the lipophilic and hydrophilic compounds analyzed, flavonoids were the most affected by PEF (due to their relative increase from 20.1 to 81.5%). This can be explained by the fact that significant amounts of these compounds are found in the outer zones of the seeds, which were more exposed to PEF. Indeed, phenolic compounds were observed predominantly in three tissues of grape seeds: epidermis, a wide part of the outer integument, and the inner layer of the inner integument [27], and the quantities found in the epidermis being significantly lower than in the outer soft seed coat. On the other hand, about 60% of the lipophilic components are found in the endosperm and embryo [47]. Since the effects of PEF depend not only on the processing parameters [16] but also on the microstructural parameters of the treated material [23,26], a better understanding of these structural changes after PEF pretreatment/treatment can be beneficial and important for process optimization and design.

## 4. Conclusions

This study showed that PEF pretreatment is a valuable technique that improves the extraction efficiency of cold pressing (oil yield and flow rate) and increases the bioactive properties of grape seed oil (concentrations of sterols, tocochromanols, and phenolic compounds). Increasing the intensity of the electric field in the range of 12.5, 14.0, and 15.6 kV/cm and a treatment duration of 15 to 30 min were particularly favorable for the extraction of tocopherols and phenolic compounds (partially localized in the upper zones of the seed tissue). In addition, compared to tocochromanols and phenolic compounds as strong lipophilic and hydrophilic antioxidants, sterols exhibited greater susceptibly to high-intensity–longer-duration treatments. Further understanding of the structural and chemical changes induced by PEF may be useful in optimizing the process and improving oil quality. In addition, the chemical composition and stability of this oil during shelf storage should also be investigated.

## Figures and Tables

**Table 1:** Experimental design and list of grape seed oil samples.

Sample Name	PEF Pretreatment of Grape Pomace Seeds			Oil Production Method
	Pulse Power (kV)	Electric Field Intensity (kV/cm)	Time (min)	
CP	–	–	–	Cold pressing
PEF1_15	40	12.5	15	
PEF1_30	40	12.5	30	
PEF2_15	45	14.0	15	
PEF2_30	45	14.0	30	
PEF3_15	50	15.6	15	
PEF3_30	50	15.6	30	

**Table 2:** Effect of PEF and cold pressing on the concentration of sterols in grape seed oil.

Sterols (mg/kg)	Sample Name						
	CP	PEF1_15	PEF1_30	PEF1_15	PEF1_30	PEF1_15	PEF1_30
Campesterol	301.7 ± 0.3 ^e^	325.6 ± 0.8 ^c^	369.8 ± 0.1 ^a^	311.7 ± 0.6 ^d^	331.9 ± 0.3 ^b^	326.9 ± 0.1 ^c^	313.0 ± 0.5 ^d^
Campestanol	11.2 ± 0.0 ^e^	18.0 ± 0.1 ^b^	11.9 ± 0.7 ^de^	13.7 ± 0.2 ^c^	18.6 ± 0.3 ^ab^	20.2 ± 0.1 ^a^	13.4 ± 0.7 ^cd^
Stigmasterol	434.0 ± 0.1 ^e^	445.0 ± 0.1 ^d^	457.8 ± 0.5 ^a^	457.5 ± 0.1 ^a^	447.7 ± 0.4 ^c^	429.9 ± 0.6 ^f^	454.0 ± 0.7 ^b^
β-Sitosterol	2599.2 ± 1.6 ^g^	2962.8 ± 1.1 ^c^	3368.4 ± 2.7 ^a^	2809.1 ± 0.1 ^e^	3039.0 ± 1.3 ^b^	2943.5 ± 0.6 ^d^	2718.5 ± 1.3 ^f^
Sitostanol	11.2 ± 0.3 ^d^	15.9 ± 0.1 ^b^	12.9 ± 0.0 ^c^	18.9 ± 0.2 ^a^	12.8 ± 0.0 ^c^	12.3 ± 0.3 ^cd^	15.7 ± 0.6 ^b^
Δ5-Avenasterol	71.3 ± 0.3 ^d^	84.0 ± 0.4 ^b^	80.0 ± 0.6 ^c^	78.2 ± 0.3 ^c^	87.5 ± 0.8 ^a^	85.0 ± 0.3 ^b^	78.5 ± 0.1 ^c^
Δ5,24-Stigmastadienol	3.5 ± 0.1 ^e^	11.1 ± 0.0 ^b^	7.4 ± 0.0 ^d^	12.4 ± 0.3 ^a^	10.3 ± 0.0 ^c^	11.6 ± 0.1 ^b^	11.3 ± 0.1 ^b^
Δ7-Stigmastenol	49.6 ± 0.1 ^e^	89.2 ± 0.5 ^b^	61.8 ± 1.0 ^d^	69.6 ± 0.1 ^c^	93.9 ± 1.3 ^a^	87.0 ± 0.1 ^b^	61.7 ± 1.6 ^d^
Δ7-Avenasterol	29.8 ± 0.1 ^f^	38.7 ± 0.1 ^b^	35.2 ± 0.0 ^d^	32.9 ± 0.3 ^e^	43.2 ± 0.1 ^a^	38.8 ± 0.1 ^b^	36.5 ± 0.3 ^c^
∑ Sterols	3511.6 ± 1.0 ^g^	3999.2 ± 0.6 ^c^	4405.3 ± 1.0 ^a^	3791.6 ± 0.6 ^e^	4095.1 ± 0.3 ^b^	3982.9 ± 0.7 ^d^	3696.4 ± 2.0 ^f^

Data are presented as mean value ± standard deviation over three replicates. ANOVA to compare data; different lowercase letters in the same column indicate significant differences between samples (Tukey’s test, *p* < 0.05).

**Table 3:** Effect of PEF and cold pressing on the concentration of tocochromanols in grape seed oil.

Tocochromanols (mg/kg)	Sample Name						
	CP	PEF1_15	PEF1_30	PEF1_15	PEF1_30	PEF1_15	PEF1_30
α-Tocopherol	44.3 ± 0.4 ^e^	45.2 ± 0.4 ^e^	54.0 ± 1.0 ^c^	50.8 ± 0.5 ^d^	56.6 ± 0.8 ^b^	51.1 ± 0.2 ^d^	65.3 ± 0.4 ^a^
γ-Tocopherol	18.5 ± 0.1 ^c^	19.0 ± 0.1 ^bc^	19.3 ± 0.2 ^bc^	19.5 ± 0.5 ^b^	19.7 ± 0.4 ^ab^	19.7 ± 0.5 ^ab^	20.6 ± 0.4 ^a^
α-Tocotrienol	102.8 ± 0.4 ^e^	103.0 ± 0.8 ^e^	119.4 ± 0.1 ^b^	111.0 ± 0.5 ^d^	118.5 ± 1.3 ^bc^	117.4 ± 0.7 ^c^	133.4 ± 0.3 ^a^
γ-Tocotrienol	138.7 ± 0.2 ^d^	139.8 ± 0.7 ^d^	144.4 ± 1.0 ^c^	148.2 ± 1.3 ^b^	150.2 ± 1.2 ^b^	150.1 ± 0.3 ^b^	153.3 ± 0.7 ^a^
Plastochromanol-8	9.3 ± 0.0 ^c^	9.5 ± 0.1 ^bc^	9.6 ± 0.1 ^bc^	9.8 ± 0.2 ^b^	9.9 ± 0.2 ^ab^	9.8 ± 0.3 ^ab^	10.3 ± 0.2 ^a^
∑ Tocochromanols	313.6 ± 0.8 ^e^	316.5 ± 0.4 ^e^	346.6 ± 0.9 ^c^	339.3 ± 1.7 ^d^	354.8 ± 2.6 ^b^	348.2 ± 1.7 ^c^	382.8 ± 1.8 ^a^

Data are presented as mean value ± standard deviation over three replicates. ANOVA to compare data; different lowercase letters in the same column indicate significant differences between samples (Tukey’s test, *p* < 0.05).

**Table 4:** Effect of PEF and cold pressing on the concentration of phenolic compounds in grape seed oil.

Phenolic Compounds	Sample Name						
	CP	PEF1_15	PEF1_30	PEF1_15	PEF1_30	PEF1_15	PEF1_30
**Nonflavonoids (µg/kg)**							
Gallic acid	147.3± 5.4 ^e^	151.5 ± 1.7 ^e^	250.2 ± 4.3 ^c^	217.9 ± 2.2 ^d^	275.1 ± 3.0 ^b^	278.7 ± 1.7 ^ab^	285.4 ± 0.8 ^a^
Hydroxybenzoic acid	322.5 ± 3.6 ^d^	328.0 ± 1.6 ^d^	377.9 ± 2.3 ^b^	364.1 ± 3.0 ^c^	383.6 ± 1.2 ^a^	387.6 ± 4.4 ^a^	387.1 ± 2.8 ^a^
*p*-Coumaric acid	170.0 ± 0.9 ^f^	191.4 ± 0.5 ^e^	220.5 ± 1.1 ^d^	223.6 ± 0.4 ^c^	225.0 ± 2.3 ^c^	323.5 ± 1.1 ^b^	346.2 ± 0.4 ^a^
Ferulic acid	111.3 ± 0.9 ^f^	111.5 ± 0.9 ^f^	121.9 ± 1.0 ^e^	125.4 ± 1.4 ^d^	130.1 ± 1.2 ^c^	139.4 ± 0.7 ^b^	159.2 ± 2.0 ^a^
∑ Nonflavonoids	751.3 ± 8.7 ^g^	782.4 ± 2.9 ^f^	970.5 ± 2.7 ^e^	931.0 ± 5.7 ^d^	1013.8 ± 5.3 ^c^	1129.1 ± 4.6 ^b^	1177.9 ± 4.8 ^a^
**Flavonoids (µg/kg)**							
(+)-Catechin	637.3 ± 3.3 ^d^	640.8 ± 2.7 ^d^	639.4 ± 2.0 ^d^	639.1 ± 2.0 ^d^	653.4 ± 3.7 ^c^	959.4 ± 4.3 ^b^	1000.0 ± 2.3 ^a^
(−)-Epicatechin	200.2 ± 4.1 ^e^	213.0 ± 9.4 ^d^	333.4 ± 5.0 ^c^	417.2 ± 1.4 ^b^	419.1 ± 1.4 ^ab^	421.0 ± 1.3 ^ab^	428.5 ± 3.4 ^a^
Procyanidin dimer B1	1388.1 ± 2.7 ^e^	1470.9 ± 2.7 ^d^	1570.0 ± 11.7 ^c^	1579.0 ± 5.7 ^c^	1582.3 ± 1.5 ^c^	2435.5 ± 10.8 ^b^	2540.1 ± 10.9 ^a^
Quercetin	62.7 ± 2.0 ^e^	104.5 ± 3.5 ^d^	127.1 ± 1.2 ^b^	107.6 ± 1.7 ^cd^	123.2 ± 1.6 ^b^	112.4 ± 2.6 ^c^	140.8 ± 1.9 ^a^
Myricetin	66.2 ± 1.3 ^c^	155.2 ± 0.7 ^b^	156.8 ± 0.6 ^b^	163.0 ± 2.4 ^a^	162.0 ± 2.4 ^a^	162.8 ± 2.1 ^a^	164.3 ± 2.0 ^a^
∑ Flavonoids	2354.4 ± 8.2 ^g^	2584.4 ± 12.1 ^f^	2826.7 ± 17.1 ^e^	2906.0 ± 5.6 ^d^	2940.0 ± 5.8 ^c^	4081.1 ± 23.9 ^b^	4273.7 ± 7.1 ^a^
**Total phenols (mg/kg)**							
	30.3 ± 0.1 ^e^	30.3 ± 0.2 ^e^	32.5 ± 0.4 ^d^	32.6 ± 0.1 ^d^	33.4 ± 0.3 ^c^	34.3 ± 0.3 ^b^	37.8 ± 0.2 ^a^

Data are presented as mean value ± standard deviation over three replicates. ANOVA to compare data; different lowercase letters in the same column indicate significant differences between samples (Tukey’s test, *p* < 0.05).

**Table 5:** Effect of PEF and cold pressing on the fatty acid composition of grape seed oil.

Fatty Acids (% of Total)	Sample Name						
	CP	PEF1_15	PEF1_30	PEF1_15	PEF1_30	PEF1_15	PEF1_30
Myristic acid (C14:0)	0.06 ± 0.00 ^a^	0.06 ± 0.00 ^a^	0.06 ± 0.00 ^a^	0.06 ± 0.00 ^a^	0.06 ± 0.00 ^a^	0.06 ± 0.00 ^a^	0.06 ± 0.00 ^a^
Palmitic acid (C16:0)	7.25 ± 0.01 ^a^	7.10 ± 0.02 ^b^	7.08 ± 0.04 ^b^	7.10 ± 0.07 ^b^	7.08 ± 0.01 ^b^	7.09 ± 0.01 ^b^	7.09 ± 0.05 ^b^
Palmitoleic acid (C16:1)	0.20 ± 0.00 ^a^	0.20 ± 0.01 ^a^	0.20 ± 0.00 ^a^	0.20 ± 0.01 ^a^	0.19 ± 0.00 ^a^	0.20 ± 0.01 ^a^	0.20 ± 0.01 ^a^
Stearic acid (C18:0)	4.50 ± 0.02 ^b^	4.54 ± 0.01 ^ab^	4.55 ± 0.01 ^a^	4.53 ± 0.02 ^ab^	4.56 ± 0.01 ^a^	4.53 ± 0.01 ^ab^	4.55 ± 0.01 ^a^
Oleic acid(C18:1)	20.30 ± 0.04 ^a^	20.43 ± 0.07 ^a^	20.39 ± 0.00 ^a^	20.35 ± 0.06 ^a^	20.33 ± 0.02 ^a^	20.35 ± 0.01 ^a^	20.31 ± 0.09 ^a^
Linoleic acid(C18:2)	67.09 ± 0.02 ^b^	67.11 ± 0.05 ^b^	67.18 ± 0.01 ^ab^	67.18 ± 0.04 ^ab^	67.20 ± 0.01 ^ab^	67.21 ± 0.02 ^a^	67.22 ± 0.01 ^a^
Linolenic acid (C18:3)	0.43 ± 0.00 ^a^	0.41 ± 0.01 ^a^	0.41 ± 0.00 ^a^	0.42 ± 0.02 ^a^	0.41 ± 0.01 ^a^	0.41 ± 0.01 ^a^	0.41 ± 0.02 ^a^
Arachidic acid (C20:0)	0.16 ± 0.00 ^a^	0.16 ± 0.00 ^a^	0.16 ± 0.01 ^a^	0.17 ± 0.00 ^a^	0.17 ± 0.00 ^a^	0.16 ± 0.00 ^a^	0.16 ± 0.00 ^a^
∑ SFA	11.97 ± 0.01 ^a^	11.85 ± 0.02 ^b^	11.85 ± 0.04 ^b^	11.85 ± 0.06 ^b^	11.86 ± 0.00 ^ab^	11.84 ± 0.02 ^b^	11.86 ± 0.06 ^ab^
∑ MUFA	20.51 ± 0.03 ^a^	20.62 ± 0.07 ^a^	20.59 ± 0.01 ^a^	20.55 ± 0.05 ^a^	20.54 ± 0.02 ^a^	20.55 ± 0.02 ^a^	20.52 ± 0.02 ^a^
∑ PUFA	67.52 ± 0.02 ^ab^	67.52 ± 0.06 ^ab^	67.59 ± 0.01 ^ab^	67.60 ± 0.02 ^ab^	67.61 ± 0.01 ^ab^	67.62 ± 0.02 ^a^	67.64 ± 0.02 ^a^

Data are presented as mean value ± standard deviation over three replicates. ANOVA to compare data; different lowercase letters in the same column indicate significant differences between samples (Tukey’s test, *p* < 0.05). Abbreviations: SFA, saturated fatty acids; MUFA, monounsaturated fatty acids; PUFA, polyunsaturated fatty acids.

## Data Availability

The original contributions presented in the study are included in the article/Supplementary Material, further inquiries can be directed to the corresponding author.

## Supplementary Materials

The following supporting information can be downloaded at: https://www.mdpi.com/article/10.3390/foods13142299/s1, Table S1: Effect of PEF and cold pressing on the concentrations of total sterols, tocochromanols and phenols: contribution of oil fraction to overall grape seed composition.
