# Supplementary material for: Effect of Pulsed Electric Field Pretreatment on the Concentration of Lipophilic and Hydrophilic Compounds in Cold-Pressed Grape Seed Oil Produced from Wine Waste
Source: Foods. 2024 Jul 22;13(14):2299. doi: 10.3390/foods13142299 (PMC11276571; doi:10.3390/foods13142299)
Supplement: Supplementary file 1 [file foods-13-02299-s001.zip › foods-3088298-supplementary.pdf]

Table S1. Effect of pulsed electric field (PEF) and cold pressing on the concentrations of sterols, tocochromanols and polyphenols: contribution of the oil fraction to the overall grape seed composition

	Sample Name						
	**CP**	**PEF1_15**	**PEF1_30**	**PEF1_15**	**PEF1_30**	**PEF1_15**	**PEF1_30**
**Total sterols (mg/kg)**	211.46 ± 0.12[g]	252.72 ± 0.04[f]	318.57 ± 0.02[a]	227.54 ± 0.05[e]	300.93 ± 0.02[b]	298.41 ± 0.05[c]	288.64 ± 0.16[d]
**Total tocochromanols (mg/kg)**	24.92 ± 0.05[f]	26.32 ± 0.03[e]	32.49 ± 0.06[c]	32.15 ± 0.13[d]	33.42 ± 0.19[b]	33.58 ± 0.13[b]	37.70 ± 0.14[a]
**Total polyphenols (mg/kg)**	1826.1 ± 6.3[f]	1917.2 ± 9.9[e]	2289.8 ± 26.6[d]	2388.1 ± 5.3[c]	2398.0 ± 30.5[c]	2570.1 ± 20.8[b]	2954.7 ± 17.8[a]

Data are presented as mean value ± standard deviation over three replicates. ANOVA to compare data; different lowercase letters in the same column indicate significant differences between samples (Tukey’s test, $p < 0.05$).
